# Supplementary material for: A taxonomic revision of Rhizophora L. (Rhizophoraceae) in Thailand
Source: PeerJ. 2024 Jun 28;12:e17460. doi: 10.7717/peerj.17460 (PMC11216214; doi:10.7717/peerj.17460)
Supplement: Supplemental Information 1 [file peerj-12-17460-s001.docx]

Herbaria

| **Herbarium Code** | **Institution / Available online:** | **Location** |
| --- | --- | --- |
| AAU | Aarhus University,  https://www.aubot.dk/search_form.php | Aarhus, Denmark |
| BK | Bangkok Herbarium, Plant Varieties Protection Office, Department of Agriculture | Chatuchak, Bangkok, Thailand |
| BKF | The Forest Herbarium, Department of National Parks, Wildlife and Plant Conservation,  https://www.dnp.go.th/botany/collections/collectionsPage.html | Chatuchak, Bangkok, Thailand |
| BM | The Natural History Museum,  https://www.nhm.ac.uk/our-science/collections/botany-collections.html | London, England, U.K. |
| BR | Meise Botanic Garden,  http://www.botanicalcollections.be | Meise, Belgium |
| CAL | Botanical Survey of India,  https://ivh.bsi.gov.in/phanerogams | Howrah, West Bengal, India |
| E | Royal Botanic Garden Edinburgh,  https://data.rbge.org.uk/search/herbarium/ | Edinburgh, Scotland, U.K. |
| G | Conservatoire et Jardin botaniques de la Ville de Genève,  http://www.ville-ge.ch/cjb/ | Genève, Switzerland |
| K (including K-W) | Royal Botanic Gardens,  http://apps.kew.org/herbcat/navigator.do | Kew, England, U.K. |
| L (including U) | Naturalis Biodiversity Center,  https://bioportal.naturalis.nl/ | Leiden, The Netherlands |
| P | Muséum National d'Histoire Naturelle,  https://science.mnhn.fr/institution/mnhn/collection/p/item/search/form?lang=en_US | Paris, France |
| US | Smithsonian Institution,  https://collections.nmnh.si.edu/search/botany/ | Washington, District of Columbia, U.S.A. |
